# Supplementary figures and images for: Positional Context of Myonuclear Transcription During Injury-Induced Muscle Regeneration
Source: Front Physiol. 2022 Mar 23;13:845504. doi: 10.3389/fphys.2022.845504 (PMC9040890; doi:10.3389/fphys.2022.845504)

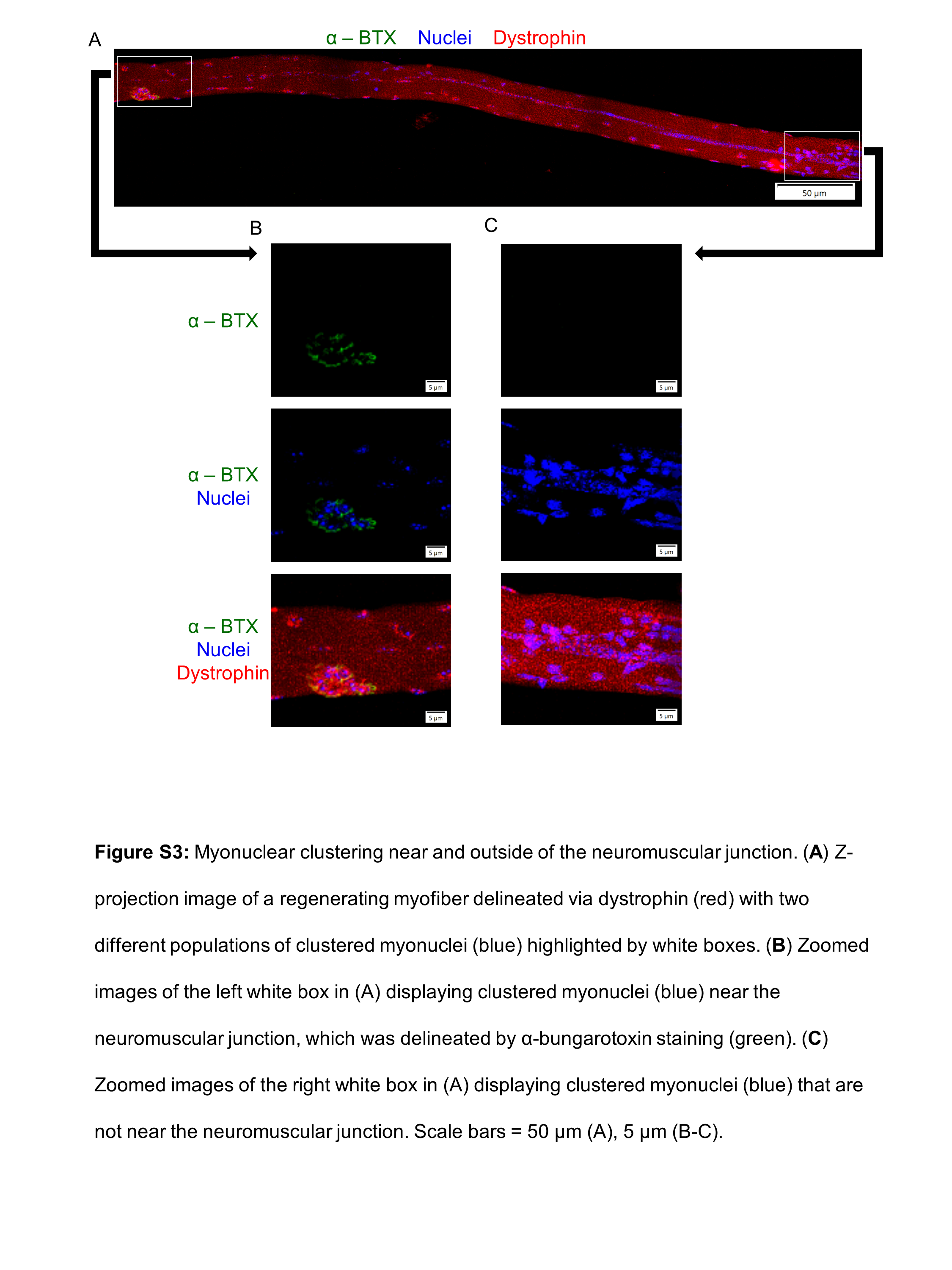

Supplement: Supplementary file 1 [file Image3.tif]

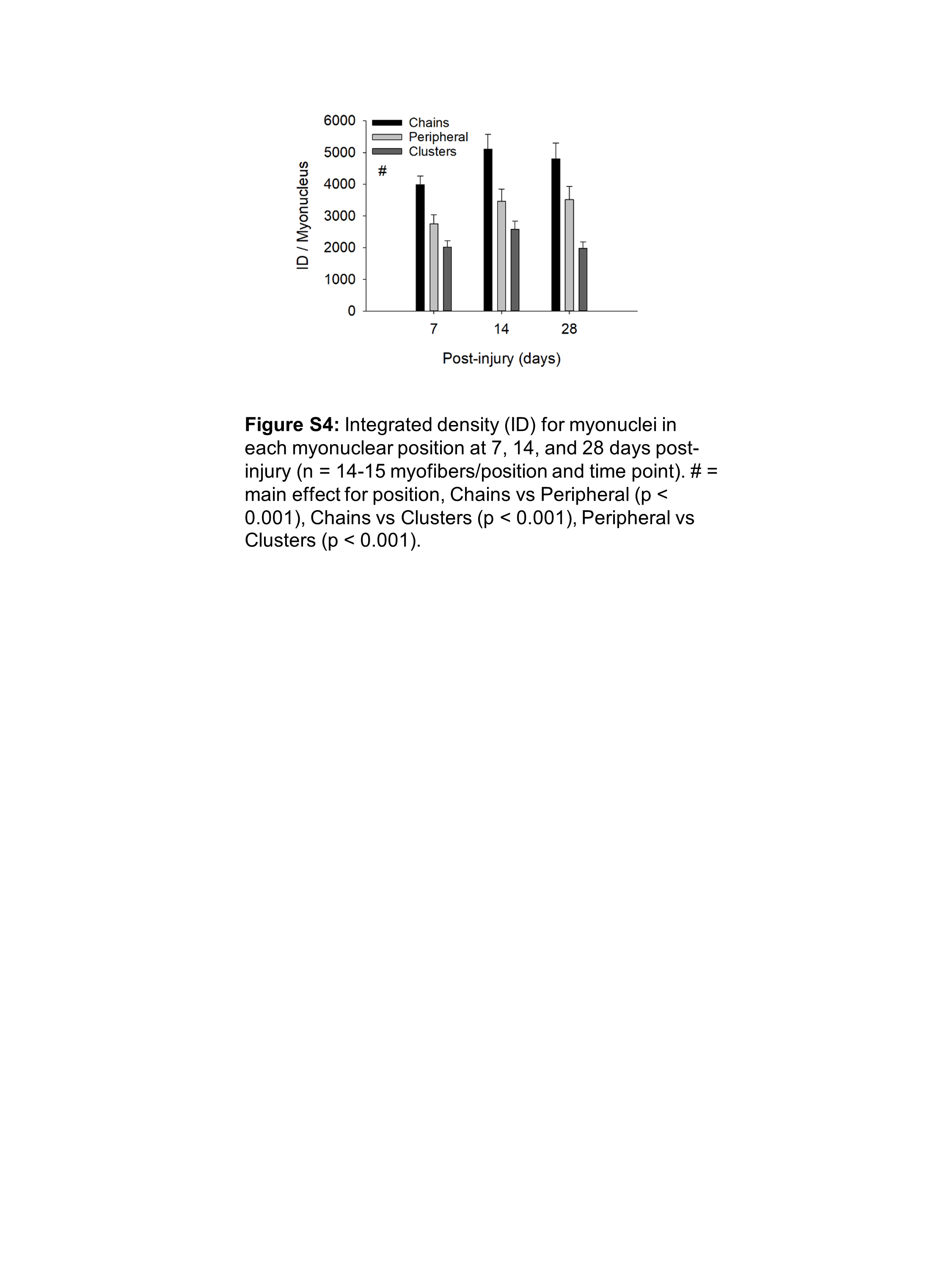

Supplement: Supplementary file 2 [file Image4.tif]

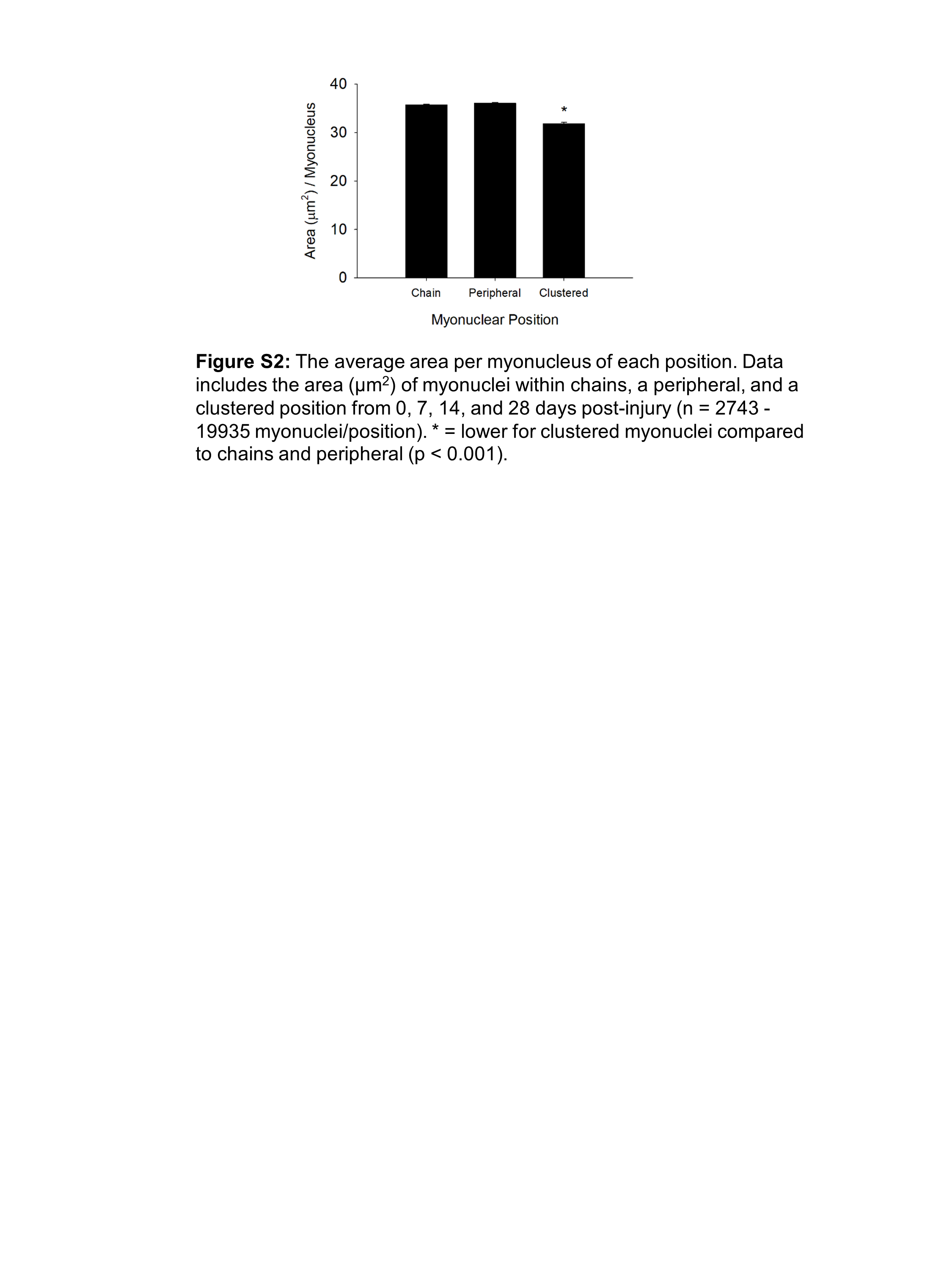

Supplement: Supplementary file 3 [file Image2.tif]

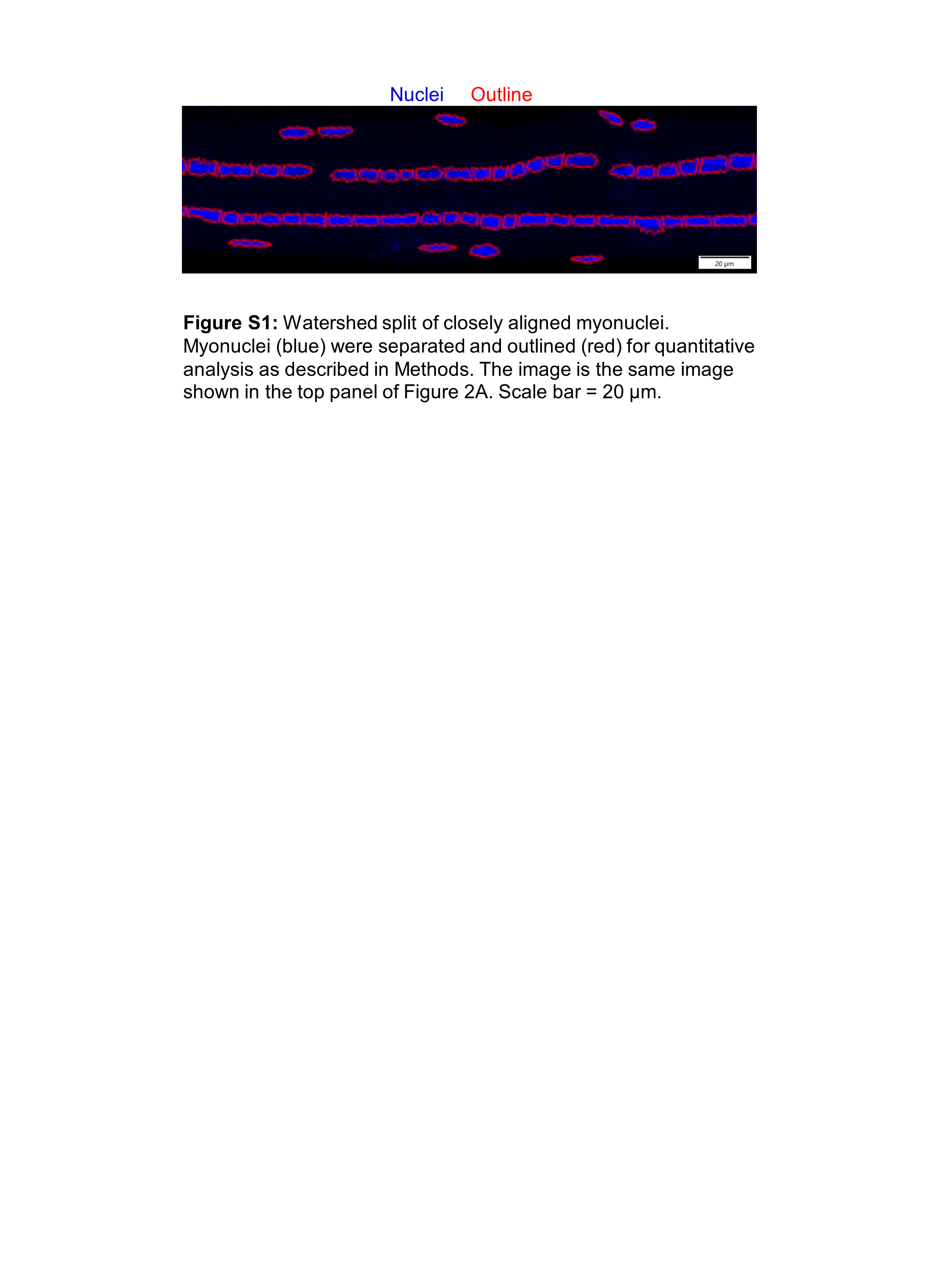

Supplement: Supplementary file 4 [file Image1.tif]

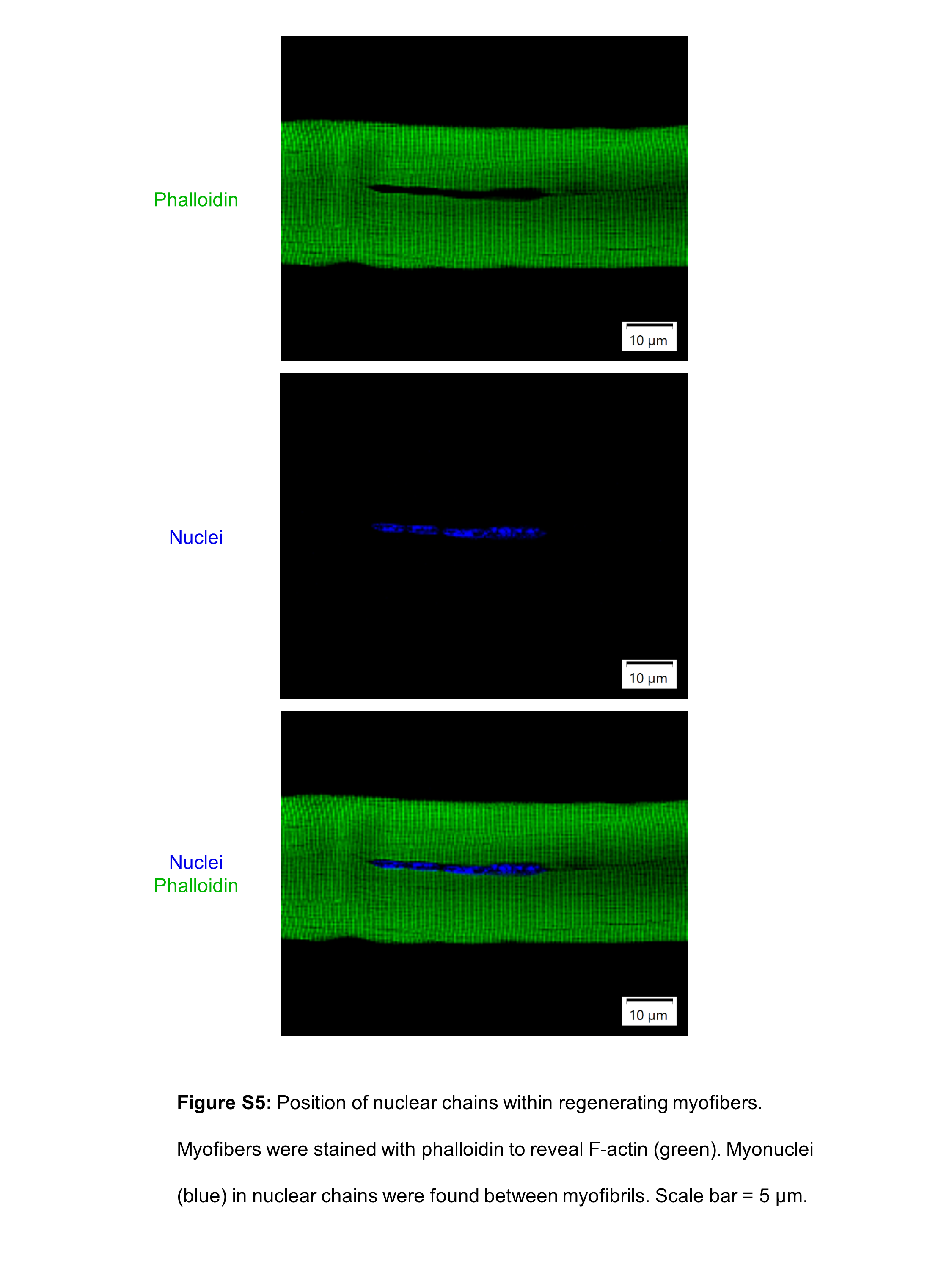

Supplement: Supplementary file 5 [file Image5.tif]
